# Diversity index as a novel prognostic factor in breast cancer

**DOI:** 10.18632/oncotarget.21371

**Published:** 2017-09-28

**Authors:** Yul Ri Chung, Hyun Jeong Kim, Young A. Kim, Mee Soo Chang, Ki-Tae Hwang, So Yeon Park

**Affiliations:** ^1^ Department of Pathology, Seoul National University College of Medicine, Seoul, Republic of Korea; ^2^ Department of Pathology, Seoul National University Bundang Hospital, Seongnam, Gyeonggi, Republic of Korea; ^3^ Department of Pathology, Seoul National University Boramae Hospital, Seoul, Republic of Korea; ^4^ Department of Surgery, Seoul National University Boramae Hospital, Seoul, Republic of Korea

**Keywords:** heterogeneity, Shannon index, c-MYC, FGFR1, copy number variation

## Abstract

Intratumoral genetic heterogeneity leads to tumor progression and therapeutic resistance. However, due to the difficulty associated with its assessment, the use of this heterogeneity as a prognostic or predictive marker remains limited. To investigate the significance of the Shannon diversity index of gene copy number variation as a tool for measuring genetic heterogeneity in breast cancer, we performed fluorescence in situ hybridization of *c-MYC* in two sets of invasive breast cancer samples and correlated the Shannon index of c*-MYC* copy number variation with clinicopathologic features and patient survival. The Shannon index was correlated with average *c-MYC* copy number and was higher in tumors in which *c-MYC* was amplified and in those with *c-MYC* genetic or regional heterogeneity. A high Shannon index was associated with adverse pathologic features including high histologic grade, lymphovascular invasion, p53 overexpression, high Ki-67 proliferation index and negative hormone receptor status. It was also associated with poor disease-free survival in the whole group, in a subgroup excluding *c-MYC-*amplified cases, and in the hormone receptor-positive subgroup of both a test and a validation set. A high Shannon index for *FGFR1* gene copy number variation was also an independent adverse prognostic factor. Our findings suggest that the Shannon diversity index is a measure of intratumoral heterogeneity and can be used as a prognostic factor in breast cancer.

## INTRODUCTION

Intratumoral heterogeneity (ITH), referring to phenotypic differences between cancer cells within the same tumor, has become a major focus of research with advances in molecular technologies. It affects important behavioral features including metastatic potential, angiogenesis, migration, evasion of antitumor immunity, and activation of metabolic pathways [1, 2]. This intratumoral diversity leads to therapeutic resistance and presents a major obstacle to cure [3].

Traditionally, genetic differences between cancer cells received most of the attention as causes of heterogeneity. However, epigenetic factors as well as environmental and stochastic factors are now also being examined. Epigenetic heterogeneity contributes even more to ITH than genetic heterogeneity, as it usually involves epigenetic silencing by DNA methylation and is enzymatically reversible [4, 5]. Environmental factors influencing heterogeneity include selection pressure on tumor cells (e.g. chemotherapy), as well as interactions with stromal cells and non-cellular elements in the tumor milieu [6, 7], while stochastic mechanisms introduce transient phenotypic variants within isogenic tumors [8]. Prevailing models of ITH include the clonal evolution model and the cancer stem cell model; rather than being mutually exclusive, these models contribute to varying extents to different tumors and create both spatial and temporal ITH [6, 9, 10].

Studies of ITH have explored somatic mutations, gene copy number alterations, and RNA expression, using both bulk tumors and single cells, comparing premalignant and malignant counterparts and multiple regions in the same tumor, as well as primary tumors and metastases [6, 11-20]. While the importance of ITH in tumors is clear, the difficulty in measuring the extent of ITH and interpreting its impact on clinical outcomes has limited its use in the clinical setting.

In a previous study, Park et al. investigated the cellular and genetic heterogeneity of breast cancers using two ecological diversity indices: the Shannon index and the Simpson index [19]. Although the Shannon index has been used in subsequent studies [6, 13, 15, 21], its prognostic significance has not been evaluated. In this study, we investigated the correlation between the Shannon index for gene copy number variation and clinicopathologic features of breast cancer, and evaluated its prognostic value in breast cancer.

## RESULTS

### c-MYC copy number variation and diversity indices

**Figure 1:**
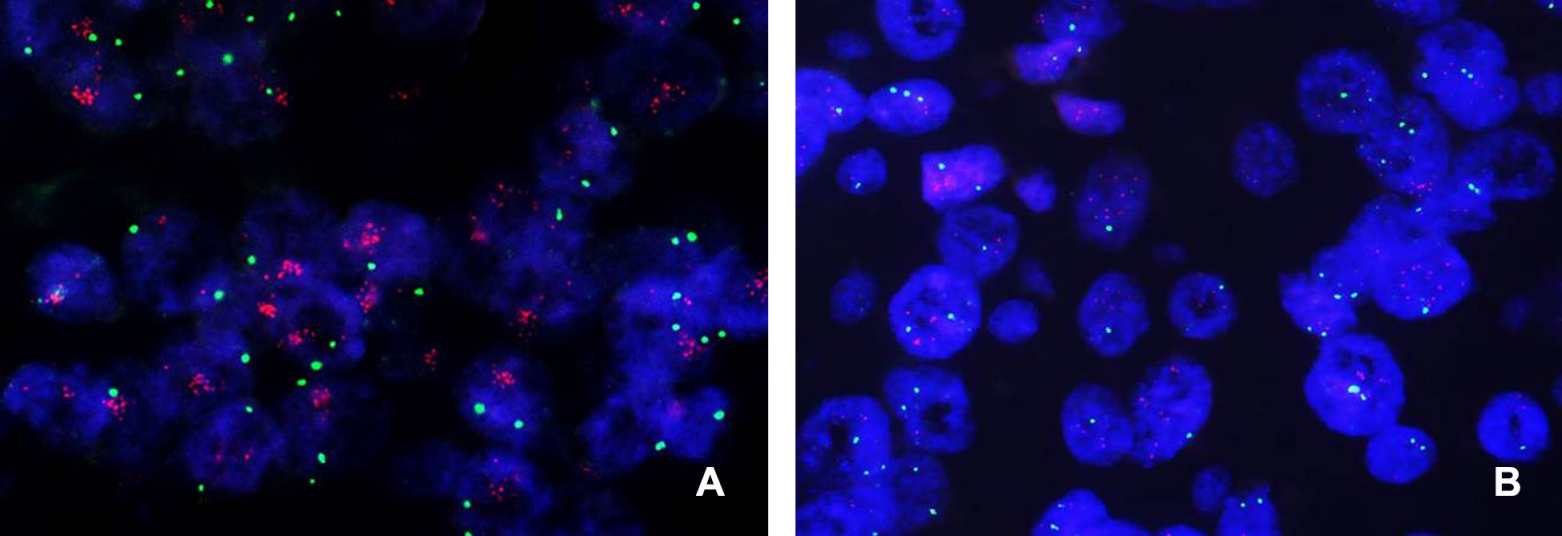
Representative images of *c-MYC* FISH in breast cancer **(A)** A case of *c-MYC* amplification with a substantially higher number of *c-MYC* (red) signals as large clusters than the normal number of centromeric (green) signals. **(B)** A case of *c-MYC* copy number gain with an increased number of three or more *c-MYC* signals per cell. Note that some cells have a copy number of greater than six.

We chose to investigate intratumoral genetic heterogeneity using *c-MYC,* since the *c-MYC* locus (8q24) is in one of the most unstable chromosomal regions and displays frequent copy number gain or amplification in all subtypes of breast cancer [22-24]. *c-MYC* amplification, defined as a mean *c-MYC* copy number of 6.0 or higher, was found in 22 (7.8%) of 283 invasive breast cancer samples in the test set (Figure 1A). *c-MYC* copy number gain, defined as a *c-MYC* copy number greater than or equal to three, was found in 115 cases (40.6%; Figure 1B). Regional heterogeneity was observed in 32 cases (11.3%), and genetic heterogeneity in 77 cases (27.2%).

**Figure 2:**
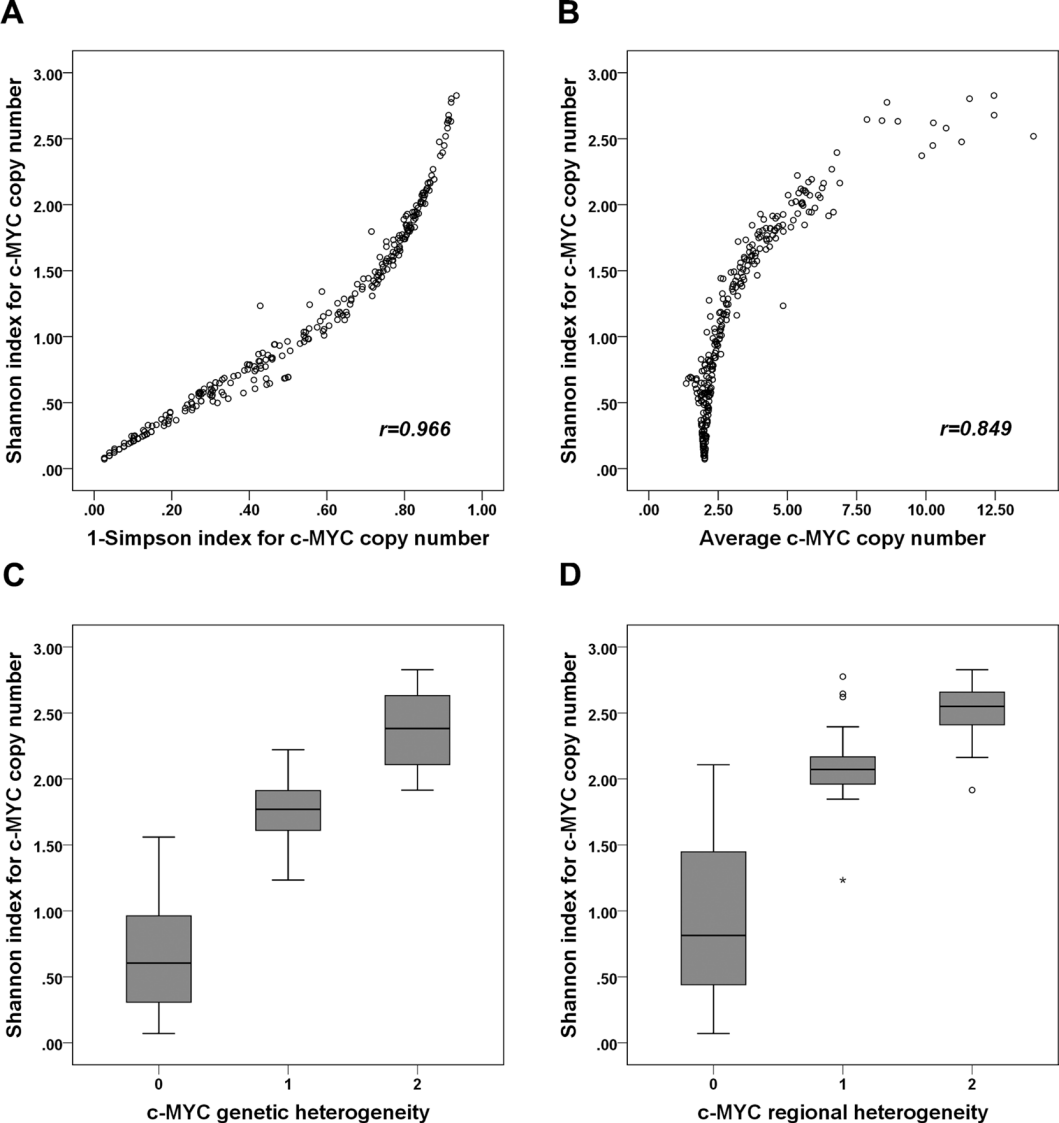
Correlation between Shannon index and 1-Simpson index, average *c-MYC* copy number, and *c-MYC* heterogeneity **(A)** The Shannon and 1-Simpson indices of *c-MYC* copy number are strongly positively correlated (*r*=0.966). **(B)** The Shannon index and average *c-MYC* copy number are also strongly positively correlated (*r*=0.849). **(C)** The Shannon index of *c-MYC* copy number is higher in tumors with genetic heterogeneity than in those with neither heterogeneity nor amplification, but it is lower than in those with amplification (without heterogeneity). **(D)** The Shannon index of *c-MYC* copy number is higher in tumors with regional heterogeneity than in those with neither regional heterogeneity nor amplification, but it tends to be lower than in those with amplification (without heterogeneity). (C, D: 0, tumors with neither heterogeneity nor amplification; 1, tumors with heterogeneity; 2; tumors with amplification without heterogeneity).

We then calculated the Shannon index of *c-MYC* copy number, which ranged from 0.071 to 2.827, with a median of 1.034. We also calculated the Simpson index and found that it ranged from 0.026 to 0.934, with a median value of 0.551. Since the two diversity indices were strongly correlated (r=0.966; p<0.001; Figure 2A), we used only the Shannon index from then on. The Shannon index was highly correlated with average *c-MYC* copy number (r=0.849; p<0.001; Figure 2B), and when we analyzed the distribution of the index with respect to *c-MYC* heterogeneity and amplification, its average was higher in tumors with genetic heterogeneity than in those with neither heterogeneity nor amplification (*p*<0.001), but it was lower than in tumors with amplification but without heterogeneity (*p*<0.001) (Figure 2C). In terms of regional heterogeneity, the index was higher in tumors with regional heterogeneity than in those that had neither heterogeneity nor amplification (*p*<0.001), and it tended to be lower than in tumors with amplification without heterogeneity (*p=*0.059) (Figure 2D).

### The association between c-MYC copy number variation and clinicopathologic features

**Figure 3:**
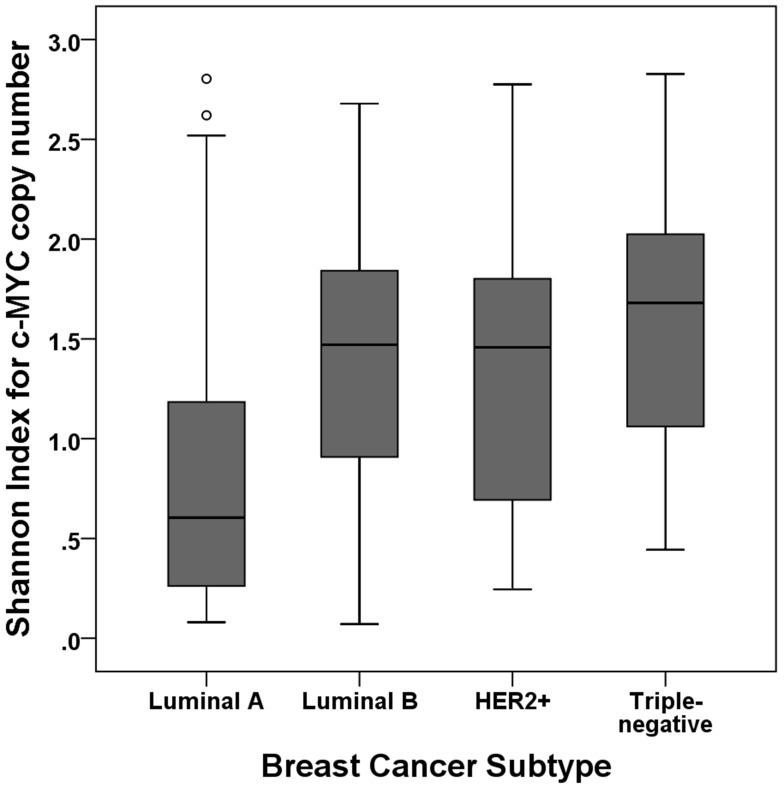
Shannon index according to subtype of breast cancer The Shannon index is significantly higher in the luminal B, HER2-positive, and triple-negative subtypes than in the luminal A subtype.

We evaluated the relationship between *c-MYC* copy number variation and clinicopathologic features (Table 1). *c-MYC* amplification was associated with high histologic grade, p53 overexpression, high Ki-67 proliferation index, and negative hormone receptor status (all *p*<0.05). *c-MYC* copy number gain was also associated with all of the clinicopathologic features associated with *c-MYC* amplification in addition to *HER2* amplification (all *p*<0.01). When we divided the samples into high index and low index groups using the median value, high Shannon index was associated with high histologic grade, lymphovascular invasion, p53 overexpression, high Ki-67 index, negative hormone receptor status, and *HER2* amplification (all *p*<0.05). The index was also significantly different according to breast cancer subtype (*p*<0.001, ANOVA test): it was significantly higher in the luminal B, HER2-positive, and triple-negative subtypes than in the luminal A subtype (*p*<0.001, *p*=0.004, *p*<0.001, respectively; Figure 3).

**Table 1:** Relationship between *c-MYC* copy number variation and clinicopathological features of tumors in the test set

Clinicopathologic characteristic	*c-MYC* amplification		*p value*	*c-MYC* copy number gain		*p value*	Shannon index for *c-MYC* copy number variation		*p value*
	Absent No. (%)	Present No. (%)		Absent No. (%)	Present No. (%)		Low No. (%)	High No. (%)	
T stage			1.000			0.578			0.206
T1-T2	245 (93.9)	21 (95.5)		159 (94.6)	107 (93.0)		136 (95.8)	130 (92.2)	
T3-T4	16 (6.1)	1 (4.5)		9 (5.4)	8 (7.0)		6 (4.2)	11 (7.8)	
N stage			0.746			0.323			0.108
N0	128 (49.0)	10 (45.5)		86 (51.2)	52 (45.2)		76 (53.5)	62 (44.0)	
N1-N3	133 (51.0)	12 (54.5)		82 (48.8)	63 (54.8)		66 (46.5)	79 (56.0)	
Histologic grade			<0.001			<0.001			<0.001
I & II	153 (58.6)	4 (18.2)		127 (75.6)	30 (26.1)		115 (81.0)	42 (29.8)	
III	108 (41.4)	18 (81.8)		41 (24.4)	85 (73.9)		27 (19.0)	99 (70.2)	
LVI			0.795			0.390			0.028
Absent	138 (52.9)	11 (50.0)		92 (54.8)	57 (49.6)		82 (59.2)	65 (46.1)	
Present	123 (47.1)	11 (50.0)		76 (45.2)	58 (50.4)		58 (40.8)	76 (53.9)	
P53 overexpression			0.002			0.004			<0.001
Absent	210 (80.5)	11 (50.0)		141 (83.9)	80 (69.6)		124 (87.3)	97 (68.8)	
Present	51 (19.5)	11 (50.0)		27 (1.1)	35 (30.4)		18 (12.7)	44 (31.2)	
Ki-67 index			0.004			<0.001			<0.001
<20%	153 (58.6)	6 (27.3)		118 (70.2)	41 (35.7)		107 (75.4)	52 (36.9)	
≥20%	108 (41.4)	16 (72.7)		50 (29.8)	74 (64.3)		35 (24.6)	89 (63.1)	
ER			0.033			<0.001			<0.001
Negative	74 (28.4)	11 (50.0)		31 (18.5)	54 (47.0)		23 (16.2)	62 (44.0)	
Positive	187 (71.6)	11 (50.0)		137 (81.5)	61 (53.0)		119 (83.8)	79 (56.0)	
PR			0.036			<0.001			<0.001
Negative	106 (40.6)	14 (63.6)		54 (32.1)	66 (57.4)		43 (30.3)	77 (54.6)	
Positive	155 (59.4)	8 (36.4)		114 (67.9)	49 (42.6)		99 (69.7)	64 (45.4)	
*HER2*			0.552			0.002			0.010
Negative	218 (83.5)	17 (77.3)		149 (88.7)	86 (74.8)		126 (88.7)	109 (77.3)	
Positive	43 (16.5)	5 (22.7)		19 (11.3)	29 (25.2)		16 (11.3)	32 (22.7)	

*P* values were calculated by the chi-square or Fisher’s exact test.

LVI, lymphovascular invasion; ER, estrogen receptor; PR, progesterone receptor; HER2, human epidermal growth factor receptor 2

### Association between Shannon index and clinical outcome

**Figure 4:**
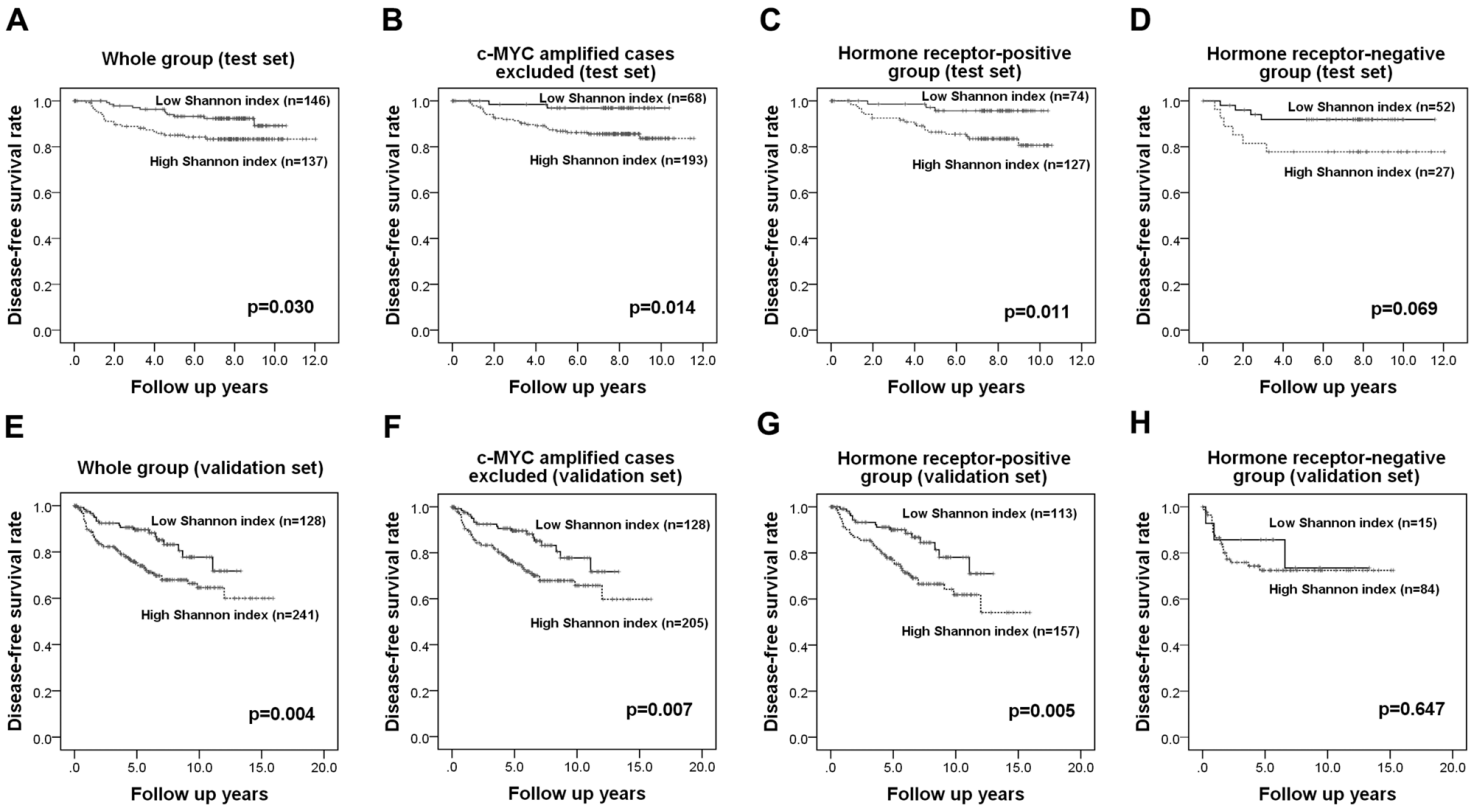
Kaplan-Meier survival analyses based on the Shannon index of *c-MYC* copy number variation In the test set, a high Shannon index is a significant adverse prognostic factor in the whole group **(A)**, in tumors without *c-MYC* amplification **(B)**, and in the hormone receptor-positive group **(C)** and shows a tendency be associated with adverse outcome in the hormone receptor-negative group **(D)**. Survival analyses in the validation set shows that the index is also a significant prognostic factor in the whole group **(E)**, in cases without *c-MYC* amplification **(F)**, and in the hormone receptor-positive subgroup **(G)** but it is not proven to be a prognostic factor in the hormone receptor-negative group **(H)**.

Most of the patients in the test set received the standard treatment and regular follow-up. The median follow-up period was 84 months (range, 1-144 months). When we analyzed the disease-free survival of patients with respect to *c-MYC* amplification and copy number gain, we found that amplification of the *c-MYC* gene was not associated with patient survival (*p*=0.324) whereas *c-MYC* copy number gain showed a tendency to be correlated with decreased disease-free survival (*p*=0.097). However a high Shannon index calculated using *c-MYC* copy number and the cutoff values obtained by ROC curve analysis revealed a significant association with poor disease-free survival (*p*=0.030, log rank test; Figure 4A). Because the Shannon index was high in tumors with *c-MYC* amplification, we performed a subgroup analysis using cases without *c-MYC* amplification to rule out the influence of *c-MYC* amplification on the Shannon index. This showed that a high Shannon index was associated with decreased survival in this subgroup as well (*p*=0.014; Figure 4B).

In univariate Cox regression analyses, high T stage (*p*=0.002), lymph node metastasis (*p*=0.002), lymphovascular invasion (*p*=0.005), and high Shannon index (*p*=0.035) correlated with an adverse clinical outcome in the whole group (Table 2). Adjuvant chemotherapy, radiation therapy, and endocrine therapy were not related to survival differences. In a multivariate analysis incorporating these covariates, only high T stage (*p*=0.024) and N stage (*p*=0.007) remained independent prognostic factors. However, when *c-MYC-*amplified cases were excluded, lymph node metastasis (*p*=0.005) and high Shannon index (*p*=0.046) also proved to be independent prognostic indicators of adverse outcome.

**Table 2:** Univariate and multivariate analyses of the association of disease-free survival in the test set with the Shannon index for *c-MYC* copy number variation

Variable	Category	Univariate analysis			Multivariate analysis		
		HR	95% CI	*P* value	HR	95% CI	*P* value
**Whole group**							
pT stage	T1-2 vs. T3-4	3.991	1.646-9.678	0.002	2.818	1.147-6.922	0.024
pN stage	N0 vs. N1-3	3.722	1.616-8.576	0.002	3.208	1.378-7.468	0.007
LVI	Absent vs. Present	3.026	1.407-6.512	0.005	1.780	0.764-4.146	0.181
Histologic grade	I & II vs. III	1.158	0.585-2.293	0.673	-	-	-
Ki-67 index	<20% vs. ≥20%	1.571	0.792-3.118	0.196	-	-	-
Hormone receptor	Positive vs. Negative	1.154	0.549-2.424	0.706	-	-	-
*HER2* amplification	Negative vs. Positive	1.932	0.898-4.158	0.092	1.571	0.717-3.440	0.259
Shannon index (*c-MYC*)	Low vs. High	2.181	1.058-4.499	0.035	1.897	0.916-3.929	0.085
**Subgroup excluding *C-MYC* amplified cases**							
pT stage	T1-2 vs. T3-4	3.413	1.300-8.956	0.013	1.972	0.736-5.283	0.177
pN stage	N0 vs. N1-3	3.817	1.554-9.376	0.003	3.676	1.496-9.031	0.005
LVI	Absent vs. Present	2.534	1.154-5.567	0.021	1.321	0.557-3.134	0.527
Histologic grade	I & II vs. III	1.142	0.549-2.376	0.722	-	-	-
Ki-67 index	<20% vs. ≥20%	1.560	0.753-3.231	0.232	-	-	-
Hormone receptor	Positive vs. Negative	1.105	0.489-2.494	0.811	-	-	-
*HER2* amplification	Negative vs. Positive	2.471	1.124-5.430	0.024	2.089	0.946-4.611	0.068
Shannon index (*c-MYC*)	Low vs. High	5.059	1.203-21.281	0.027	4.359	1.029-18.471	0.046
**Hormone receptor-positive subgroup**							
pT stage	T1-2 vs. T3-4	3.083	0.915-10.391	0.069	1.605	0.464-5.557	0.455
pN stage	N0 vs. N1-3	4.211	1.432-12.381	0.009	3.860	1.311-11.366	0.014
LVI	Absent vs. Present	2.410	0.991-5.860	0.052	1.313	0.505-3.411	0.576
Histologic grade	I & II vs. III	1.307	0.554-3.084	0.541	-	-	-
Ki-67 index	<20% vs. ≥20%	2.091	0.916-4.770	0.080	1.454	0.621-3.405	0.389
*HER2* amplification	Negative vs. Positive	1.727	0.587-5.079	0.321	-	-	-
Shannon index (*c-MYC*)	Low vs. High	4.269	1.268-14.370	0.019	3.862	1.145-13.023	0.029

HR, hazard ratio; LVI, lymphovascular invasion; HER2, human epidermal growth factor receptor 2; CI, confidence interval

Since the Shannon index also varied greatly according to hormone receptor status, we also performed a subgroup analysis by hormone receptor status using cut-off values based on ROC curve analysis in each subgroup. In the hormone receptor-positive group, a high Shannon index was correlated with a worse prognosis (*p*=0.011, log rank test; Figure 4C) while in the hormone receptor-negative group, it only showed a tendency to be correlated with poor clinical outcome (*p*=0.069, log rank test; Figure 4D). In a multivariate Cox regression analysis (Table 2), high N stage (*p*=0.014), and high Shannon index (*p*=0.029) proved to be independent predictive factors for poor clinical outcome in the hormone receptor-positive group.

### Prognostic performance of the Shannon index of c-MYC copy number variation in a validation set

In a validation set of 369 invasive breast cancers, high Shannon index for *c-MYC* copy number variation also correlated with adverse clinicopathologic features including high histologic grade, lymphovascular invasion, p53 overexpression, high Ki-67 proliferation index, and negative hormone receptor status (all *p*<0.001; Supplementary Table 1). The median patient follow-up period was 72 months (range, 1-191 months), and there were no survival differences with regard to post-operative treatment modalities. In survival analyses, high Shannon index was associated with decreased disease-free survival in the whole group and in the subgroup excluding *c-MYC-* amplified cases (*p*=0.004, *p*=0.007, respectively, log-rank test; Figure 4E-4F). In subgroup analyses by hormone receptor status, a high Shannon index was associated with poor patient survival in the hormone receptor-positive group, but not in the hormone receptor-negative group (*p*=0.005, *p*=0.647, respectively; Figure 4G-4H). In addition to the Shannon index for *c-MYC* copy number, high T stage, lymph node metastasis, and lymphovascular invasion were found to be significant prognostic factors in univariate analyses. However, in multivariate analysis, Shannon index was not found to be an independent prognostic factor for disease-free survival in the whole group, in the subgroup excluding *c-MYC-*amplified cases, or in the hormone receptor-positive subgroup (Supplementary Table 2).

### Validation of the prognostic impact of the Shannon index using FGFR1

**Figure 5:**
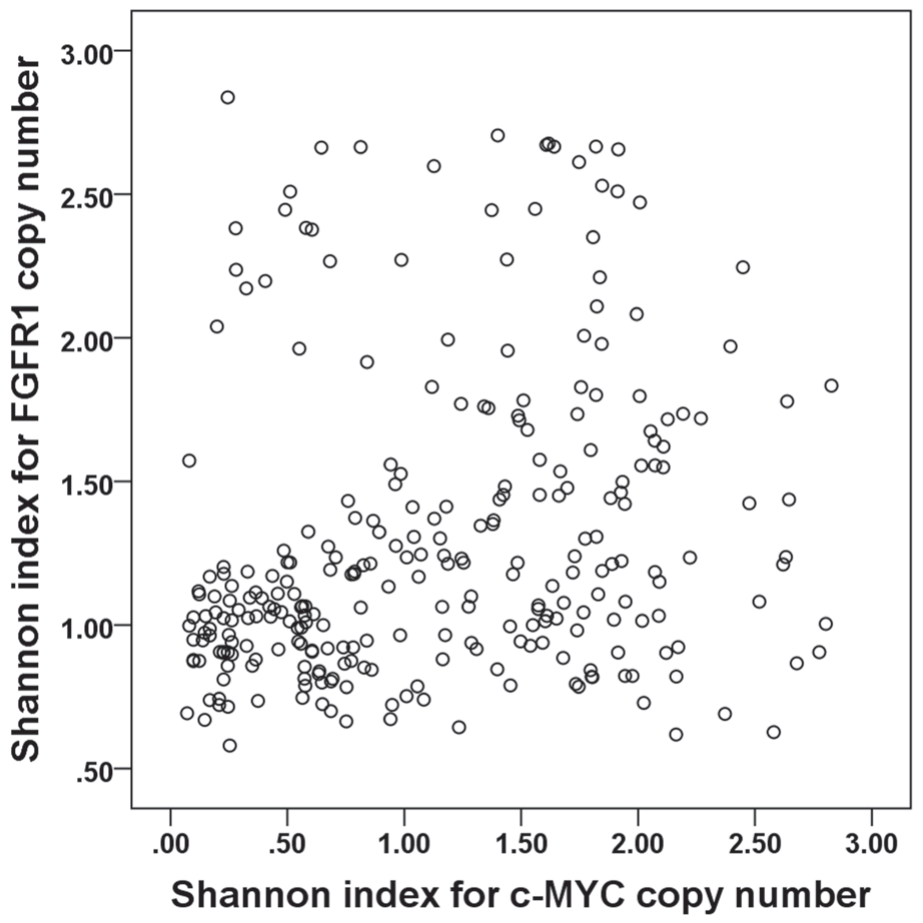
Correlation between Shannon indices of *c-MYC* and *FGFR1* copy number variation The Shannon indices for *c-MYC* and *FGFR1* copy number variations are positively correlated (*r*=0.233).

**Figure 6:**
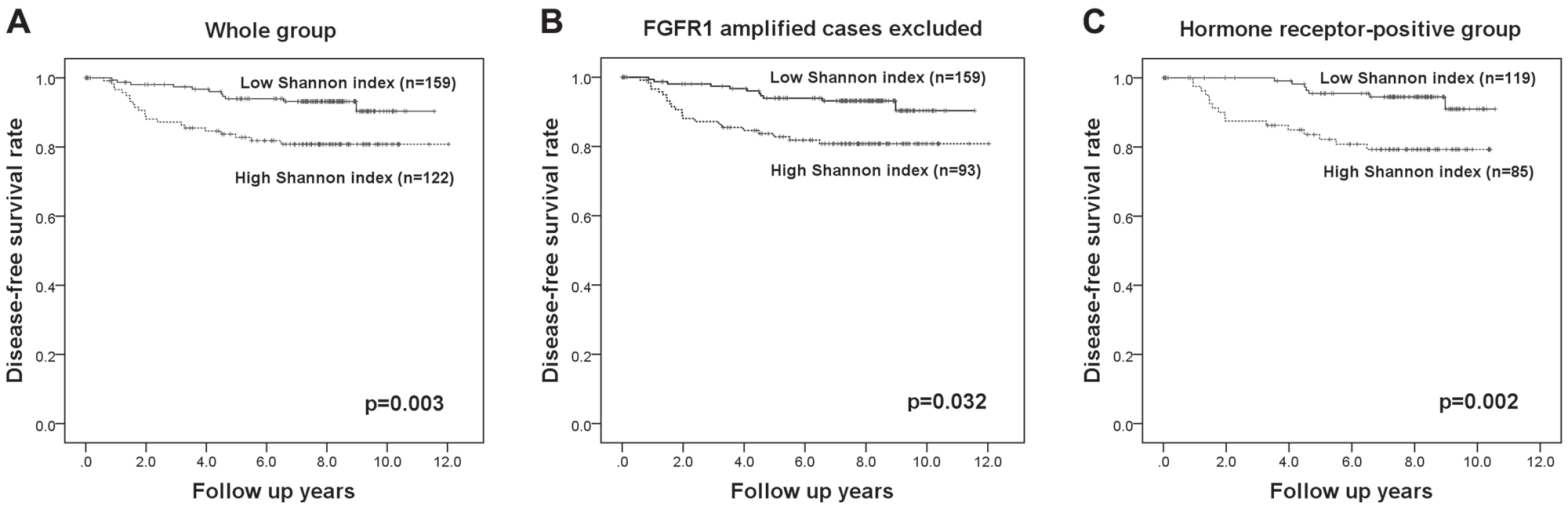
Kaplan-Meier survival analyses based on the Shannon index of *FGFR1* copy number variation High Shannon indices for *FGFR1* copy number variation are associated with poor disease-free survival in the whole group **(A)**, in those without *FGFR1* amplification **(B)**, and in the hormone receptor-positive subgroup **(C)**.

We wondered whether the Shannon index for a different gene would also have significance as a prognostic factor. We therefore followed the same procedures in the test set with *FGFR1* as we did with *c-MYC*. *FGFR1* copy number variation was assessed in 281 of the 283 invasive breast cancers by FISH (all the tissue microarray cores has been lost in two cases). *FGFR1* amplification was not significantly associated with any clinicopathologic features of breast cancer, but p53 overexpression and *HER2* amplification tended to be elevated in tumors with *FGFR1* amplification (*p*=0.089, *p*=0.085, respectively; Supplementary Table 3). A high Shannon index of *FGFR1* copy number was correlated with high histologic grade and p53 overexpression (*p*=0.044, *p*=0.007, respectively; Supplementary Table 3), and the Shannon indices for *c-MYC* and *FGFR1* were correlated (r=0.233; *p*<0.001; Figure 5). *FGFR1* amplification is a well-known adverse prognostic factor in breast cancer, especially in hormone receptor-positive cases [25-27], and we found that *FGFR1* amplification was correlated with decreased disease-free survival in the whole group and in the hormone receptor-positive subgroup, but not in the hormone receptor-negative subgroup (*p*=0.003, *p*=0.009, *p*=0.143, respectively). A high Shannon index for *FGFR1* copy number variation was also correlated with decreased disease-free survival (*p*=0.003; Figure 6A). Again, when *FGFR1*-amplified cases were excluded, high Shannon index for *FGFR1* copy number variation was associated with poor clinical outcome (*p*=0.032; Figure 6B). In a subgroup analysis by hormone receptor status, high Shannon index for *FGFR1* copy number variation was correlated with poor clinical outcome in the hormone receptor-positive subgroup (*p*=0.002; Figure 6C) but not in the receptor-negative subgroup (*p*=0.532).

In multivariate analyses (Table 3), high Shannon index proved to be an independent poor prognostic factor in the whole group as well as in the subgroup excluding *FGFR1*-amplified cases and in the hormone receptor-positive subgroup (*p*=0.003, *p*=0.028, *p*=0.005, respectively).

**Table 3:** Univariate and multivariate analyses of the association of disease-free survival in the test set with the Shannon index for FGFR1 copy number variation

Variable	Category	Univariate analysis			Multivariate analysis		
		HR	95% CI	*P* value	HR	95% CI	*P* value
**Whole group**							
pT stage	T1-2 vs. T3-4	3.991	1.646-9.678	0.002	3.221	1.299-7.990	0.012
pN stage	N0 vs. N1-3	3.722	1.616-8.576	0.002	3.390	1.446-7.945	0.005
LVI	Absent vs. Present	3.026	1.407-6.512	0.005	1.732	0.747-4.012	0.200
Histologic grade	I & II vs. III	1.158	0.585-2.293	0.673	-	-	-
Ki-67 index	<20% vs. ≥20%	1.571	0.792-3.118	0.196	-	-	-
Hormone receptor	Positive vs. Negative	1.154	0.549-2.424	0.706	-	-	-
*HER2* amplification	Negative vs. Positive	1.932	0.898-4.158	0.092	2.021	0.936-4.362	0.073
Shannon index (*FGFR1*)	Low vs. High	2.844	1.379-5.866	0.005	3.041	1.472-6.316	0.003
**Subgroup excluding *FGFR1*-amplified cases**							
pT stage	T1-2 vs. T3-4	5.596	2.231-14.038	<0.001	4.550	1.779-11.639	0.002
pN stage	N0 vs. N1-3	3.987	1.496-10.624	0.006	3.334	1.227-9.059	0.018
LVI	Absent vs. Present	3.143	1.313-7.526	0.010	1.687	0.647-4.401	0.285
Histologic grade	I & II vs. III	1.368	0.624-2.998	0.434	-	-	-
Ki-67 index	<20% vs. ≥20%	1.716	0.779-3.781	0.180	-	-	-
Hormone receptor	Positive vs. Negative	1.226	0.529-2.842	0.634	-	-	-
*HER2* amplification	Negative vs. Positive	2.345	0.979-5.621	0.056	2.194	0.907-5.311	0.081
Shannon index (*FGFR1*)	Low vs. High	2.312	1.050-5.094	0.038	2.430	1.102-5.360	0.028
**Hormone receptor-positive subgroup**							
pT stage	T1-2 vs. T3-4	3.083	0.915-10.391	0.069	1.609	0.460-5.622	0.457
pN stage	N0 vs. N1-3	4.211	1.432-12.381	0.009	4.223	1.436-12.423	0.009
LVI	Absent vs. Present	2.410	0.991-5.860	0.052	1.261	0.484-3.286	0.635
Histologic grade	I & II vs. III	1.307	0.554-3.084	0.541	-	-	-
Ki-67index	<20% vs. ≥20%	2.091	0.916-4.770	0.080	1.954	0.856-4.460	0.112
HER2 amplification	Negative vs. Positive	1.727	0.587-5.079	0.321	-	-	-
Shannon index (*FGFR1)*	Low vs. High	3.710	1.526-9.021	0.004	3.600	1.480-8.759	0.005

HR, hazard ratio; LVI, lymphovascular invasion; HER2, human epidermal growth factor receptor 2; CI, confidence interval

## DISCUSSION

Understanding intratumoral heterogeneity (ITH) is critical for both cancer research and treatment, since it ultimately leads to tumor progression. Previous studies in breast cancer have investigated intratumoral genetic heterogeneity using somatic mutations, gene copy number alterations, mRNA expression, and microRNA expression [11, 13, 15, 16, 18, 21, 28-30]. While the vast majority of such studies confirmed the presence of ITH, only a few studies investigated its impact on response to treatment or patient prognosis. Of the factors affected by ITH in breast cancer, *HER2* heterogeneity can be easily evaluated in breast cancer and can be matched with the response to *HER2*-targeted therapy, and thus has been the subject of many investigations including one by our group [31-33]. It is accepted that *HER2* heterogeneity is associated with poor clinical outcome and therapeutic resistance to *HER2*-targeted agents such as trastuzumab. However, in general, measurement of intratumoral genetic heterogeneity is complicated, and thus, its application as a biomarker representing disease progression and therapeutic resistance has been limited. To our knowledge, this is the first study investigating the use of the index as an indicator of tumor progression in breast cancer.

We conducted this study using the *c-MYC* gene, one of the genes most frequently amplified across all cancer types [34], and we validated our results using a different set of tumor samples from another institution and another frequently-amplified gene, *FGFR1*. We found that a high Shannon index of *c-MYC* copy number variation was associated with poor disease-free survival in both the test and validation sets, and we obtained similar results after excluding cases in which *c-MYC* was amplified, so ruling out the effect of *c-MYC* amplification on the diversity index. Using *FGFR1* in place of *c-MYC* for calculating the diversity index confirmed the generality of these results.

In the previous study that quantified the degree of ITH of breast cancer using the Shannon diversity index [19], the authors discovered that a high degree of genetic heterogeneity existed not only between distinct tumor cell populations but also between the tumor cells within the same population. While it was suggested that a larger tumor cell population size, and hypoxia, might increase intratumoral genetic diversity, the association between the Shannon index and the histopathologic features was not statistically significant due to the small sample size. In the present study, we were able to show that the histopathological features associated with aggressive tumor behaviors (high histologic grade, lymphovascular invasion, p53 overexpression, high Ki-67 index, and negative hormone receptor status) were significantly associated with a high diversity index for *c-MYC* copy number variation in both a test set and a validation set. As *c-MYC* dysregulation promotes chromosomal instability [35], the diversity index for *c-MYC* copy number variation may reflect the degree of chromosomal instability. Therefore, the association between high diversity index and aggressive features of breast cancer is in line with the results from a previous study by Endesfelder et al. who found that chromosomal instability in breast cancer was associated with high histologic grade and hormone receptor negativity [36]. The Shannon index based on *FGFR1* copy number variation, on the other hand, was not associated with any of those clinicopathologic features, and a possible explanation is that the diversity index for copy number variation of a given gene represents the effects of both amplification of that gene and genetic heterogeneity for that gene. In this study, *FGFR1* amplification was not associated with any clinicopathologic features of breast cancer, but it tended to show an association with p53 overexpression. Accordingly, the Shannon index for *FGFR1* copy number variation was significantly associated with high histologic grade and p53 overexpression.

Recently, Almendro et al. evaluated ITH using the Shannon index in primary and metastatic breast cancer samples using seven different genes [21]. They found that the extent of intratumoral genetic diversity in a tumor was similar regardless of the chromosomal region analyzed, suggesting that it may be an inherent property of a tumor. We also observed that the Shannon diversity indices for *c-MYC* and *FGFR1* copy number variation were correlated, again suggesting that the degree of ITH in a tumor is an intrinsic feature of that tumor. Almendro et al. investigated the genetic and phenotypic diversity of primary tumors, and matched lymph nodes and distant metastases, and found that the diversity was greatest among the distant metastases; we also plan to perform a study of tumor progression comparing primary and metastatic tumors to clarify the significance of the association between high diversity index and disease progression.

We observed that a high Shannon index using *c-MYC* copy number was an adverse prognostic factor in the hormone receptor-positive subgroup, but not in the hormone receptor-negative subgroup in both the test and validation sets. Similarly, a high Shannon index based on *FGFR1* copy number variation was an independent poor prognostic factor only in the hormone receptor-positive subgroup. This result may be explained by the different extents of genomic instability in different subtypes of breast cancer. Kwei et al. divided the genetic alterations in breast cancer into three types: a “simple” type harboring only a few copy number alterations characteristic of the luminal A subtype, an “amplifier” type with focal high-level DNA amplification in the luminal B and HER2 subtypes, and, lastly, a “complex” type characteristic of the triple-negative subtype [37]. In our subgroup analysis, the hormone receptor-negative group included HER2-positive and triple-negative tumors; these subtypes usually show high levels of genomic instability and have high diversity indices, which may be the reason that the Shannon diversity index had no prognostic value in this subgroup. On the other hand, in the hormone receptor-positive group, a high diversity index may reflect the contribution of the luminal B subtype, which has a higher level of genomic instability than the luminal A subtype. However, we have observed that even when a survival analysis was restricted to the luminal A subtypes, a high diversity index had prognostic significance (data not shown).

There are some limitations to this study. First, although the treatment per se did not affect the survival of patients, this was a retrospective study and the patients were not treated uniformly. A large-scale prospective study in a uniformly-treated patient population may be needed to confirm the prognostic value of the diversity index. Second, we counted gene signals per cell by FISH using tissue section, which inevitably includes truncation artifacts that may lead to artificial heterogeneity. The diversity index measured in this study included some artificial heterogeneity, but this was true for all of the cases. Lastly, our analysis was confined to the *c-MYC* and *FGFR1* genes. Although we showed that high diversity indices for *c-MYC* and *FGFR1* gene copy number variation were associated with adverse clinical outcomes, it is not clear that this association is true for other genes. This warrants further study.

To conclude, we have shown that high Shannon indices of *c-MYC* and *FGFR1* copy number variation are associated with adverse features of breast cancer. A high diversity index is also a significant prognostic factor for decreased patient survival. Thus it appears that the Shannon diversity index is a measure of ITH and a prognostic factor in breast cancer that can identify those at high risk of recurrence or progression, and it can be used in the clinical setting for deciding the optimal treatment.

## MATERIALS AND METHODS

### Patients and tissue samples

Our test set consisted of 283 invasive breast cancer samples that had been resected consecutively from 2003 to 2007 at Seoul National University Bundang Hospital. We validated our results using 369 cases of invasive breast cancer resected at Seoul National University Boramae Hospital between 1999 and 2012. Recurrent breast cancers, advanced breast cancers with distant metastasis at presentation, and cases with incomplete resection, were excluded. Clinicopathologic data were collected from electronic medical records and pathology reports. The following histopathologic variables were recorded: tumor size, T stage, N stage, histologic subtype (by WHO classification), histologic grade (by the Bloom and Richardson grading system), lymphovascular invasion, estrogen receptor (ER), progesterone receptor (PR) and HER2 status, Ki-67 proliferation index, and p53 overexpression. The baseline characteristics of the test set and validation set are listed in Supplementary Table 4. This study was approved by the Institutional Review Board (protocol # B-1601/332-304), and informed consent was waived.

### Tissue microarray construction

All of the slides from surgically-resected specimens in the test set and the validation set were reviewed, and three representative regions were selected. In tumors that showed different histologic features, areas with different histologies were chosen. We constructed tissue microarrays (TMAs) of 2mm diameter from each of these regions, yielding three cores per case (SuperBioChips Laboratories, Seoul, South Korea) for immunohistochemistry and fluorescence in situ hybridization.

### Fluorescence in situ hybridization assays for c-MYC and FGFR1

Fluorescence in situ hybridization (FISH) for *c-MYC* was performed with the following commercially available locus-specific probes and chromosome enumeration probes (CEPs): LSI *c-MYC* SpectrumOrange probe (8q24.12-q24.13) and CEP 8 SpectrumGreen probe (8p11.1-q11.1) (Abbott Molecular, Downers Grove, IL, USA). Fibroblast growth factor receptor 1 (*FGFR1*) FISH was performed with locus-specific BAC, RP11-100B16 (chr8:38,358,839-38,522,417) and CEP 8 SpectrumGreen probes (8p11.1-q11.1) (Abbott Molecular). We obtained the BAC clone from Invitrogen (Carlsbad, CA, USA) and purified it with a large construction kit (Qiagen, Valencia, CA, USA). DNA from the BAC clone was labeled with SpectrumOrange using a nick translation kit (Abbott Molecular).

Briefly, 4-μm deparaffinized TMA cores were incubated in pretreatment solution (Abbott Molecular) at 80°C for 30 min followed by protease solution (Abbott Molecular) for 20 min at 37°C. The probes were diluted in tDen-Hyb-2 hybridization buffer (InSitus Biotechnologies, Albuquerque, NM, USA). DNA denaturation of the probes and the tissue sections was achieved by incubating them in HYBriteTM (Abbott Molecular) for 5 min at 73°C followed by hybridization at 37°C for 16 hours. Post-hybridization washes were performed according to the manufacturer’s instructions. Slides were mounted in 4, 6-diamidino-2-phenylindole/anti-fade and viewed under a fluorescence microscope.

Gene signals per cell in 50 tumor nuclei were evaluated for each TMA core: 150 tumor cells were thus counted in each case. Average gene copy number was calculated separately for each TMA core and in combination. Gene amplification was considered to be present when the average gene copy number of the three TMA cores was 6.0 or higher, and copy number gain was defined as an average gene copy number of ≥3 in the three TMA cores. Cases that showed both amplification and non-amplification depending on the core were considered to have regional heterogeneity. Following the guidelines for defining *HER2* genetic heterogeneity [38], cases in which the proportion of cells with amplification was between 5 and 50% were considered to be genetically heterogeneous. The presence of regional and genetic heterogeneity was assessed, and the degree of heterogeneity was evaluated using two diversity indices as described below.

### Diversity indices

The Shannon index is a diversity index that quantifies the uncertainty in assigning the species identity of an individual in a population, and is a popular index in ecology. It is calculated as

H’ = -∑ p_i_ ln(p_i_), where p_i_ equals the frequency of species i in the population [39]. A species, in this study, represents those tumor cells with the same copy number of *c-MYC* or *FGFR1*. As pointed out in a previous study [19], one of the shortcomings of the Shannon index is a tendency to confound species richness and evenness, and therefore we calculated the Simpson index for comparison. The Simpson index (D=∑ p_iz_) is another well-known ecological index; it has the advantage that it has a clear biological and probabilistic interpretation but it has the disadvantage that the most abundant species contribute disproportionately to the value obtained [19, 40, 41]. We computed the Simpson index along with Shannon index in our initial analysis.

### Definition of breast cancer subtypes

Immunohistochemical expression of the standard biomarkers that had been evaluated in whole sections at the time of diagnosis (and during the study in cases with missing data) were used to categorize the tumor samples into breast cancer subtypes according to the 2011 St. Gallen Expert Consensus [42] as follows: luminal A (ER+ and/or PR+, HER2-, Ki-67<14%), luminal B (ER+ and/or PR+, HER2-, Ki-67≥14%; ER+ and/or PR+, HER2+), HER2+ (ER-, PR-, HER2+), and triple-negative subtype (ER-, PR-, HER2-). ER and PR expression was measured in 10% increments, and 1% or more stained nuclei were considered positive. For HER2, 3+ on immunohistochemistry or the presence of gene amplification in FISH was considered positive.

### Statistical analysis

We analyzed our data using Statistical Package, SPSS version 22.0 for Windows (SPSS Inc, Chicago, IL, USA). Correlations between Shannon index, Simpson index, and *c-MYC* copy number were evaluated by Pearson’s correlation test. The median value of the Shannon index was used as a cutoff point for assigning tumors into the low or high Shannon index categories, and the associations between Shannon index and clinicopathologic features of the tumors were evaluated by the Chi-square test or Fisher’s exact test. Differences in Shannon index between multiple groups were analyzed by one-way analysis of variance (ANOVA) and the Turkey post hoc test. A receiver operating characteristic (ROC) curve analysis was performed to identify the cut-off values of the Shannon index that maximized the sum of sensitivity and specificity in predicting clinical outcomes. Disease-free survival was analyzed by drawing Kaplan-Meier curves, and differences were determined with the log-rank test. Multivariate analysis was performed with a Cox proportional hazard regression model using a backward stepwise selection method using the covariates significantly associated with patient outcome in the univariate analyses. Hazard ratios (HR) and their 95% confidence intervals (CI) were calculated for each variable. *P*-values < 0.05 were considered statistically significant. All *p*-values were two-sided.

## SUPPLEMENTARY MATERIALS TABLES




