# Supplementary material for: Diversity index as a novel prognostic factor in breast cancer
Source: Oncotarget. 2017 Sep 28;8(57):97114–26. doi: 10.18632/oncotarget.21371 (PMC5722549; doi:10.18632/oncotarget.21371)
Supplement: Supplementary file 1 [file oncotarget-08-97114-s001.pdf]

Diversity index as a novel prognostic factor in breast cancer

SUPPLEMENTARY MATERIALS

Supplementary Table 1: Relationship between Shannon index for *c-MYC* copy number variation and clinicopathological features of tumors in the validation set

Clinicopathologic characteristic	Shannon index for *c-MYC* copy number variation		*p* value
	Low	High	
	No. (%)	No. (%)	
T stage			0.524
T1-T2	165 (89.7)	162 (87.6)	
T3-T4	1 (10.3)	23 (12.4)	
N stage			0.156
N0	109 (59.2)	96 (51.9)	
N1-N3	75 (40.8)	89 (48.1)	
Histologic grade			<0.001
I & II	152 (82.6)	80 (43.2)	
III	32 (17.3)	105 (56.8)	
LVI			<0.001
Absent	134 (72.8)	94 (50.8)	
Present	50 (27.2)	91 (49.2)	
P53 overexpression			<0.001
Absent	158 (85.9)	107 (57.8)	
Present	26 (14.1)	78 (42.2)	
Ki-67 index			<0.001
<20%	145 (78.8)	75 (40.5)	
≥20%	39 (21.2)	110 (59.5)	
ER			<0.001
Negative	32 (17.4)	92 (49.7)	
Positive	152 (82.6)	93 (50.3)	
PR			<0.001
Negative	55 (29.9)	92 (49.7)	
Positive	129 (70.1)	93 (50.3)	
HER2			0.094
Negative	154 (83.7)	142 (76.8)	
Positive	30 (16.3)	43 (23.2)	

P values were calculated by the chi-square or Fisher's exact test.
LVI, lymphovascular invasion; ER, estrogen receptor; PR, progesterone receptor; HER2, human epidermal growth factor receptor 2

Supplementary Table 2: Univariate and multivariate analyses of the association of disease-free survival in the validation set with Shannon index for *c-MYC* copy number variation

Variable	Category	Univariate analysis			Multivariate analysis		
		HR	95% CI	*P* value	HR	95% CI	*P* value
**Whole group**							
pT stage	T1-2 vs. T3-4	3.106	1.882-5.124	<0.001	2.047	1.215-3.447	0.007
pN stage	N0 vs. N1-3	3.338	2.115-5.268	<0.001	2.860	1.760-4.647	<0.001
LVI	Absent vs. Present	2.438	1.593-3.731	<0.001	1.426	0.888-2.291	0.142
Histologic grade	I & II vs. III	1.385	0.906-2.115	0.132	-	-	-
Ki-67 index	<20% vs. ≥20%	1.344	0.878-2.055	0.173	-	-	-
Hormone receptor	Positive vs. Negative	1.183	0.739-1.895	0.484	-	-	-
HER2 amplification	Negative vs. Positive	1.007	0.599-1.692	0.979	-	-	-
Shannon index (*c-MYC*)	Low vs. High	2.064	1.241-3.434	0.005	1.656	0.988-2.776	0.056
**Subgroup excluding *C-MYC* amplified cases**							
pT stage	T1-2 vs. T3-4	2.976	1.751-5.056	<0.001	1.976	1.137-.3.432	0.016
pN stage	N0 vs. N1-3	3.437	2.103-5.618	<0.001	2.750	1.635-4.623	<0.001
LVI	Absent vs. Present	2.171	1.382-3.413	0.001	1.258	0.761-2.076	0.370
Histologic grade	I & II vs. III	1.289	0.811-2.047	0.283	-	-	-
Ki-67 index	<20% vs. ≥20%	1.319	0.834-2.088	0.237	-	-	-
Hormone receptor	Positive vs. Negative	1.256	0.758-2.079	0.376	-	-	-
HER2 amplification	Negative vs. Positive	0.908	0.516-1.598	0.738	-	-	-
Shannon index (*c-MYC*)	Low vs. High	2.015	1.198-3.387	0.008	1.577	0.928-2.680	0.092
**Hormone receptor-positive subgroup**							
pT stage	T1-2 vs. T3-4	2.991	1.618-5.529	<0.001	2.046	1.090-3.842	0.026
pN stage	N0 vs. N1-3	3.687	2.115-6.427	<0.001	3.307	1.869-5.850	<0.001
LVI	Absent vs. Present	2.110	1.271-3.483	0.003	1.255	0.721-2.185	0.421
Histologic grade	I & II vs. III	1.388	0.816-2.362	0.226	-	-	-
Ki-67 index	<20% vs. ≥20%	1.361	0.805-2.300	0.250	-	-	-
HER2 amplification	Negative vs. Positive	1.141	0.608-2.141	0.681	-	-	-
Shannon index (*c-MYC*)	Low vs. High	2.202	1.249-3.885	0.006	1.588	0.884-2.854	0.122

HR, hazard ratio; LVI, lymphovascular invasion; HER2, human epidermal growth factor receptor 2; CI, confidence interval

Supplementary Table 3: Relationship between Shannon index for *FGFR1* copy number variation and clinicopathological features of tumors in the test set

Clinicopathologic characteristic	*FGFR1* amplification		*p* value	Shannon index for *FGFR1* copy number variation		*p* value
	Absent	Present		Low	High	
	No. (%)	No. (%)		No. (%)	No. (%)	
T stage			1.000			0.409
T1-T2	236 (93.7)	28 (96.6)		128 (92.8)	136 (95.1)	
T3-T4	16 (6.3)	1 (3.4)		10 (7.2)	7 (4.9)	
N stage			0.766			0.854
N0	123 (48.8)	15 (51.7)		67 (48.6)	71 (49.7)	
N1-N3	129 (51.2)	14 (48.3)		71 (51.4)	72 (50.3)	
Histologic grade			0.664			0.044
I & II	141 (56.0)	15 (51.7)		85 (61.6)	71 (49.7)	
III	111 (44.0)	14 (48.3)		53 (38.4)	72 (50.3)	
LVI			0.171			0.750
Absent	138 (54.8)	12 (41.4)		75 (54.3)	75 (52.4)	
Present	114 (45.2)	17 (58.6)		63 (45.7)	48 (47.6)	
P53 overexpression			0.089			0.007
Absent	200 (79.4)	19 (65.5)		117 (84.8)	102 (71.3)	
Present	52 (20.6)	10 (34.5)		21 (15.2)	41 (28.7)	
Ki-67			0.577			0.237
<20%	144 (57.1)	15 (51.7)		83 (60.1)	76 (53.1)	
≥20%	108 (42.9)	14 (48.3)		55 (39.9)	67 (46.9)	
ER			0.774			0.171
Negative	76 (30.2)	8 (27.6)		36 (26.1)	48 (33.6)	
Positive	176 (69.8)	21 (72.4)		102 (73.9)	95 (66.4)	
PR			0.365			0.120
Negative	109 (43.3)	10 (34.5)		52 (37.7)	67 (46.9)	
Positive	143 (56.7)	19 (65.5)		86 (62.3)	76 (53.1)	
HER2			0.085			0.104
Negative	214 (84.9)	21 (72.4)		120 (87.0)	114 (79.7)	
Positive	38 (15.1)	8 (27.6)		18 (13.0)	29 (20.3)	

LVI, lymphovascular invasion; ER, estrogen receptor; PR, progesterone receptor; HER2, human epidermal growth factor receptor 2.

Supplementary Table 4: Baseline characteristics.

See Supplementary File 1
