# Supplementary material for: Diversity index as a novel prognostic factor in breast cancer
Source: Oncotarget. 2017 Sep 28;8(57):97114–26. doi: 10.18632/oncotarget.21371 (PMC5722549; doi:10.18632/oncotarget.21371)
Supplement: Supplementary file 2 [file oncotarget-08-97114-s002.pdf]

Supplementary Table 4: Baseline characteristics

Characteristic	No (%)	
	Test set	Validation set
Age, yrs.		
Mean	49	53
Range	26-87	25-87
T stage		
T1	126 (44.5)	124 (33.6)
T2	140 (49.5)	203 (55.0)
T3	8 (2.8)	27 (7.3)
T4	9 (3.2)	15 (4.1)
N stage		
N0	138 (48.8)	205 (55.6)
N1	83 (29.3)	102 (27.6)
N2	40 (14.1)	36 (9.8)
N3	22 (7.8)	26 (7.0)
Histologic subtype		
Invasive ductal carcinoma	247 (87.3)	325 (88.1)
Invasive lobular carcinoma	14 (4.9)	18 (4.9)
Mucinous carcinoma	9 (3.2)	7 (1.9)
Others	13 (4.6)	19 (5.1)
Histologic grade		
I	65 (23.0)	50 (13.6)
II	92 (32.5)	182 (49.3)
III	126 (44.5)	137 (37.1)
Lymphovascular invasion		
Absent	149 (52.7)	228 (61.8)
Present	134 (47.3)	141 (38.2)
P53 overexpression		
Negative	221 (78.1)	265 (71.8)
Positive	62 (21.9)	104 (28.2)
Ki-67 index		
<20%	159 (56.2)	220 (59.6)
≥20%	124 (43.8)	149 (40.4)
ER		
Negative	85 (30.0)	124 (33.6)
Positive	198 (70.0)	245 (66.4)
PR		
Negative	120 (42.4)	147 (39.8)
Positive	163 (57.6)	222 (60.2)
HER2		
Negative	235 (83.0)	296 (80.2)
Positive	48 (17.0)	73 (19.8)
Subtype		
Luminal A	140 (49.5)	163 (44.2)
Luminal B	64 (22.6)	107 (29.0)
HER2-positive	26 (9.2)	27 (7.3)
Triple-negative	53 (18.7)	72 (19.5)
Adjuvant chemotherapy		
Received	217 (76.7)	296 (80.2)
Not received	60 (21.2)	71 (19.2)
Unknown	6 (2.1)	2 (0.5)
Adjuvant radiation therapy		
Received	138 (48.8)	153 (41.5)
Not received	139 (49.1)	214 (58.0)
[illegible]	[illegible]	[illegible]

Adjuvant endocrine therapy		
Received	194 (68.6)	223 (60.4)
Not received	83 (29.3)	125 (33.9)
Unknown	6 (2.1)	21 (5.7)

ER, estrogen receptor; PR, progesterone receptor; HER2, human epidermal growth factor receptor 2
